# Modeling the Attractor Landscape of Disease Progression: a Network-Based Approach

**DOI:** 10.3389/fgene.2017.00048

**Published:** 2017-04-18

**Authors:** Atefeh Taherian Fard, Mark A. Ragan

**Affiliations:** Institute for Molecular Bioscience, University of Queensland, St. Lucia, QLD, Australia

**Keywords:** attractor, landscape, disease progression, gene-regulatory networks, Hopfield networks

## Abstract

Genome-wide regulatory networks enable cells to function, develop, and survive. Perturbation of these networks can lead to appearance of a disease phenotype. Inspired by Conrad Waddington's epigenetic landscape of cell development, we use a Hopfield network formalism to construct an attractor landscape model of disease progression based on protein- or gene-correlation networks of Parkinson's disease, glioma, and colorectal cancer. Attractors in this landscape correspond to normal and disease states of the cell. We introduce approaches to estimate the size and robustness of these attractors, and take a network-based approach to study their biological features such as the key genes and their functions associated with the attractors. Our results show that the attractor of cancer cells is wider than the attractor of normal cells, suggesting a heterogeneous nature of cancer. Perturbation analysis shows that robustness depends on characteristics of the input data (number of samples per time-point, and the fraction which converge to an attractor). We identify unique gene interactions at each stage, which reflect the temporal rewiring of the gene regulatory network (GRN) with disease progression. Our model of the attractor landscape, constructed from large-scale gene expression profiles of individual patients, captures snapshots of disease progression and identifies gene interactions specific to different stages, opening the way for development of stage-specific therapeutic strategies.

## Introduction

Gene regulatory networks (GRNs) regulate diverse biological processes including cell-lineage commitment and differentiation. GRNs are robust in maintaining their functionality against a wide range of perturbations. Inappropriate regulatory signals can trigger cascades of failures that cause GRNs to malfunction and a disease phenotype to appear (Huang et al., 2009; del Sol et al., 2010). In most instances it is not aberrant activity of a single gene, but rather the perturbation of gene networks, that drives disease progression (Stower, 2012).

Computational modeling provides insight into GRN dynamics and how the interplay of genes can lead to alternative phenotypes. One modeling framework is the attractor landscape. Huang et al. (2005) described a high-dimensional space in which each coordinate represents the expression of a gene in the GRN. Attractors in this landscape correspond to stable equilibrium states associated with a specific cell type (Huang et al., 2005, 2009). Disease phenotypes such as cancer can be viewed as abnormal cell types and represented as latent attractors in this landscape (Huang et al., 2009). Trajectories across the landscape correspond to developmental processes or disease progression, and elevation (i.e., the z-axis) is inversely proportional to the likelihood of a particular state (GRN configuration; Huang et al., 2009; Wang et al., 2010).

Experimental studies have confirmed that attractor landscapes and state-space trajectory models can provide insight into the biological basis of developmental processes (Huang et al., 2005; Chang et al., 2008; Huang, 2009). Other frameworks have been employed to model such state-space trajectories and attractor landscapes, including model-free approaches (Chen et al., 2012); *logical models* such as Boolean networks and Petri nets, which provide a quantitative model of a GRN, allowing users to gain an overall understanding of the behavior of the system under different conditions; and *continuous models* such as those based on ordinary differential equations (ODEs) or continuous linear models, which provide a framework to capture and understand stochasticity in the system using real-valued molecular concentrations (rather than discretized values) over a continuous time scale (Karlebach and Shamir, 2008). In the context of cancer, Saez-Rodriguez et al. (2011) used logical models to compare normal and transformed hepatocyte networks; Esfahani et al. (2011) proposed an algorithm based on Boolean networks with perturbation, and through partial knowledge of the GRN and gene-expression values reconstructed tumor progression; and Lucia and Maino (2002) used ODEs to model the interaction of tumors with the host immune system. These frameworks tend to be based on small gene regulatory circuits, and/or require extensive prior knowledge of the system (Maetschke and Ragan, 2014; Taherian Fard et al., 2016).

If disease is viewed as a pre-existing configuration of the GRN, accessed via specific mutations or other changes to the system (Huang et al., 2009), one can model trajectories of disease progression by using an appropriate time-course gene-expression profile. However, given the scarcity of homogenous or isogenic samples (as samples come from different patients), lack of dynamic experimental data, and very limited time-course disease progression gene-expression data, to date it has not been feasible to construct a comprehensive model of GRN dynamics in disease.

Here we employ the mathematical formalism of Hopfield networks (HNs; Hopfield, 1982) to construct and visualize the landscape of disease progression, based on large-scale gene-expression profiles from patients with different stages of disease or cancer grades. We characterize normal and disease states of the cell as attractors of Hopfield networks; estimate their size and robustness; and take a network-based approach to identify the unique biomolecular interactions that underlie each stage of disease progression. These attractors correspond to local minima of an energy function, and are formed by iteratively updating the network; transient states correspond to intermediate time-points in the gene-expression profile, while trajectories trace the convergence of samples to their attractors. We hypothesize that an attractor with a large basin is more likely to attract a more-heterogeneous set of samples. To remain as close to the biology as possible, we utilize a correlation network (computed from large-scale time-course gene-expression data) to construct the model. Changes in this correlation network through time correspond to rewiring of the GRN through the stages of disease progression.

## Materials and methods

### Hopfield networks

**Figure 1:**
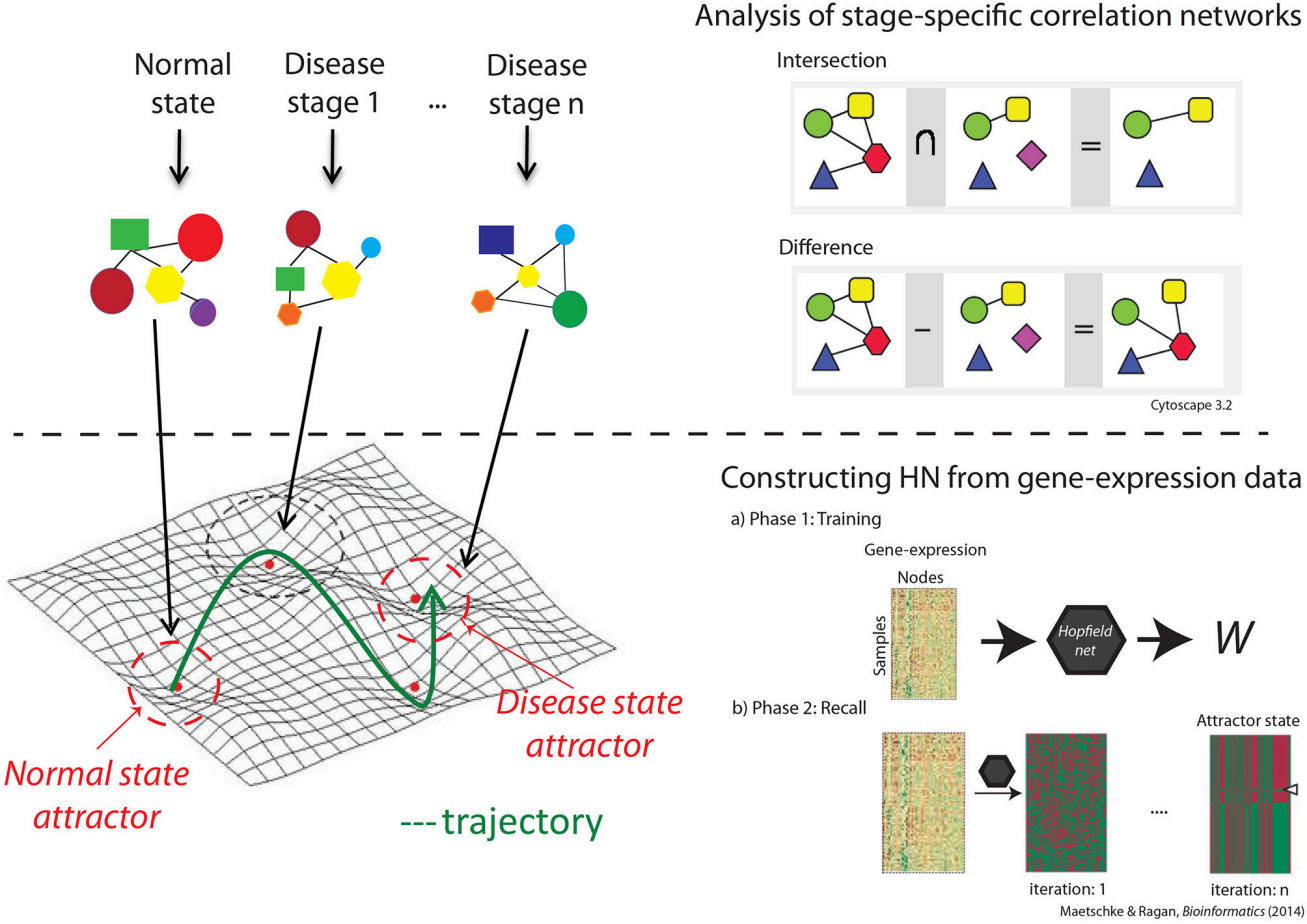
**The workflow**. The top panel describes the correlation network analysis through which we identified unique genes and interactions at each disease stage, and biological characteristics of stage-specific correlation networks. The bottom panel shows the steps taken in constructing the network through which we visualized the landscape and estimated the size and robustness of the attractors.

We used Hopfield networks (HNs; Hopfield, 1982) to model trajectories of disease progression and track rewiring of the underlying GRN. An HN is a fully connected neural network with nodes *i* ∈ 1,…, *n* and undirected edges *w*_*ij*_ between nodes *i* and *j*, representing genes and their interaction, respectively. There are two major steps involved in constructing the HN. Firstly in the training phase, we construct the weight matrix *W* based on the Pearson correlation coefficient (PCC) between the gene pairs. *W* is a symmetric matrix, with *w*_*ij*_ = PCC (*i,j*) and *w*_*ii*_ = 0 for nodes *i* and *j*. Secondly, in the recall phase, dynamics of the network and convergence to the attractors are defined by the product of the pattern matrix (gene expression profile) and the weight matrix *W* (bottom panel in Figure 1).

$$\begin{array}{r} P_{\left(t+1\right)}=sgn\left(P_{\left(t\right)}W\right) \end{array}$$

where *P*_(*t*)_ is the state of the pattern (here samples) at time step *t*, followed by a discretization through a sign function.

The energy *E* is computed through a family of monotonically decreasing functions, in this case the Lyapunov function that guarantees convergence to a low-energy attractor state.

$$\begin{array}{r} E\left[P\left(s\right)\right]=-\frac{1}{2}PWP^{T} \end{array}$$

where *E*[*P*(*s*)] is the energy of the network state *s* at time *t*, for pattern *P*.

We do not provide sample labels to the algorithm; the pattern learning step is non-parametric, that is we leave it to the algorithm to construct attractors based on similarities among the patterns. To visualize the landscape in three dimensions, we interpolated the energy values over a two-dimensional grid constructed from the first and the second principal components of the dimensionally reduced gene-expression data. For more details please refer to Taherian Fard et al. (2016). This work differs from and extends our previous methods as follows: (1) here the term “attractor” refers to a Hopfield attractor that is generated as the result of an iteration process and corresponds to a local minimum of the energy function; (2) the perturbation analysis is carried out directly on the *W* by assigning random values to the edges in the network; and (3) we introduce methods to measure the width and depth of attractors. All HN analysis were performed on a standard workstation (Windows OS) and completed in <5 s for each dataset.

### Estimating the size and robustness of an attractor

We estimated the size of attractors by measuring their width and depth. To estimate the width of an attractor, we computed the intra-group distance of all the samples converging to the attractor. There are different approaches to calculate the intra-group distance of the elements in a group, including sum, minimum, maximum or average pairwise distance between all points in a group, or between the centroid and all points in the cluster. To obtain a comparable measure of width across all attractors we used the average standardized pair-wise Euclidean distance of samples converging to the same attractor. This estimate provides a quantitative measure of the variation of samples converging to a specific attractor. The depth was measured by calculating the energy difference of samples before and after convergence.

Although the elements of the GRN remain the same, their interactions change from normal to disease phenotype. In our model, the *W* matrix holds information on the interactions between the entities of the network. Therefore, in order to assess the effect of perturbations on the network, we randomly perturbed 50% of the edges in *W*. From our previous study (Taherian Fard et al., 2016) we know that at 50% perturbation, the network does not reflect a stable phenotype, but enough signal remains (i.e., has not been randomized away) consistent with the network representing a transient or unstable cell state in a living system. The *W* matrix perturbation step was then followed by the HN recall phase using the perturbed *W*, computation of the fraction of the samples that did not converge to their respective attractor, and computation of the Hamming distance (HD) of the samples to their attractor after each iteration. The HD between the two binary strings indicates the proportion of values that disagree between them. The highest difference is observed at HD = 1. At HD = 0, the strings are identical. The Hamming distance was computed using SciPy tool 0.15.0 (http://www.scipy.org) in Python.

### Datasets

The first case-study (DeMarshall et al., 2015) identifies candidate blood-based autoantibody biomarkers that are useful for early detection and diagnosis of Parkinson's disease (PD). ProtoArray v5.0 Human Protein Microarrays (Invitrogen, Carlsbad CA) were used to identify differentially expressed autoantibodies in human serum samples. With an overall accuracy of 97.5%, the candidate biomarkers were capable of distinguishing early-stage PD from advanced PD. The dataset (GEO accession number: GSE62283) encompasses 45 human serum samples, 15 for each disease stage: control (normal), early PD (EPD), and advanced PD patients (Table S1).

The second case-study (Sun et al., 2006) investigates the effect of stem cell factor (SCF) expression in human gliomas in a grade-dependant manner. SCF induces angiogenic response *in vivo* by directly activating brain microvascular endothelial cells. Sun et al. (2006) found that SCF overexpression is associated with poor prognosis in glioma patients, whereas its down-regulation results in improved survival in mouse models. SCF is up-regulated in high-grade gliomas and down-regulated in non-tumor samples, making it a potential anti-angiogenic target in malignant gliomas. The dataset (GEO accession number: GSE4290) includes mRNA expression data (Affymetrix Human Genome U133 Plus 2.0 Array) from patients with different grades of glioma. The 96 samples include 23 non-tumor samples (from epilepsy patients), 38 grade II, 12 grade III, and 23 grade IV gliomas (Table S1).

The third case-study compares the gene-expression profiles of primary tumors with and without distant metastasis, to identify candidate genes that influence the prognosis of patients with colorectal cancer (CRC). Using real-time reverse transcription PCR, Matsuyama et al. (2010) found that grade II and grade III CRC patients with low expression of MUC12 showed the worst disease-free survival, suggesting prognostic value for MUC12 in postoperative adjuvant therapy for these patients. There are 17 normal (from adjacent tissues), 47 non-metastatic, and 30 metastatic samples in this dataset (GEO accession number: GSE18105; Table S1).

Each dataset was *z*-score normalized (μ = 0, σ = ±1), followed by feature selection to extract probes with the highest variation across groups. We ranked genes based on their variance. The index beyond which the variance is essentially unchanged (that is, the elbow of the variance-over-feature plot) was used as a cut-off to select the number of features (genes).

### Biological properties of stage-specific networks

Correlation network were constructed for each disease stage, and common genes and interactions across groups were identified. We used the first 100 feature-selected genes to construct the correlation networks, and chose the genes with significant (*p* < 0.0001) interactions for further analysis. The *p*-values were generated using standard *t* statistics and were generated as a significance measure while computing the PPC. We subtracted the set of common genes and interactions from the original networks to obtain a network of genes with unique interactions specific to each disease stage (top panel in Figure 1).

We used Ingenuity Pathway Analysis (QIAGEN, Redwood City CA) for gene function and biological network analyses. Statistical analysis was performed using R 3.0.1. Correlation network comparisons and visualization were performed using Cytoscape 3.2.1 (Shannon et al., 2003).

## Results

**Figure 2:**
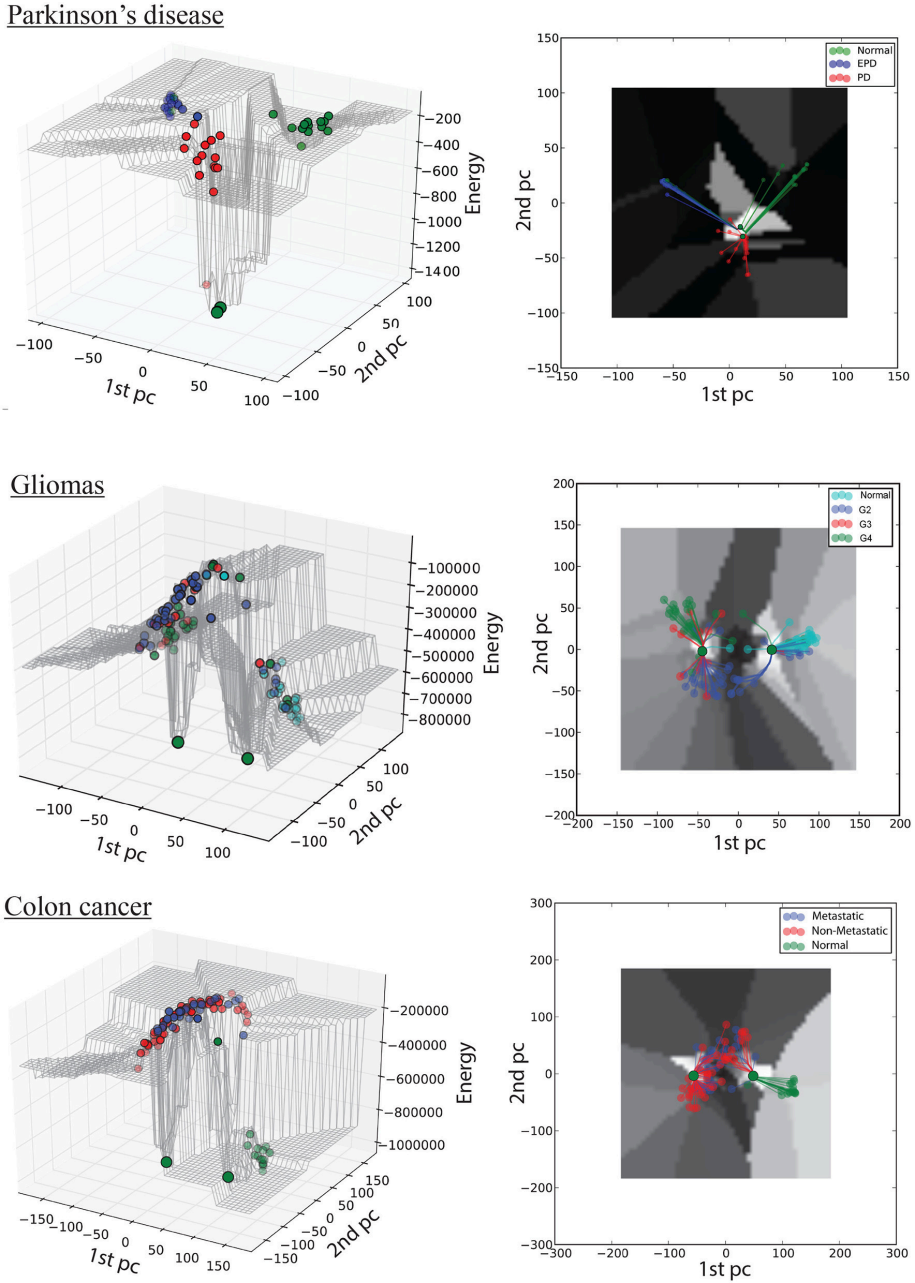
**Hopfield energy landscape for the three case studies in 3D (left column) and 2D (right column): top, Parkinson's disease; middle, gliomas; bottom panel, colon cancer**. The *x-* and *y-*axes are respectively the first and the second principal components of the autoantibody expression data, while the *z-*axis is the Hopfield energy value. Each dot represent a sample, colored by disease stage; and the large green dots represent the attractors. The lines in the 2D view represent the trajectories of samples converging to their respective attractors.

**Figure 3:**
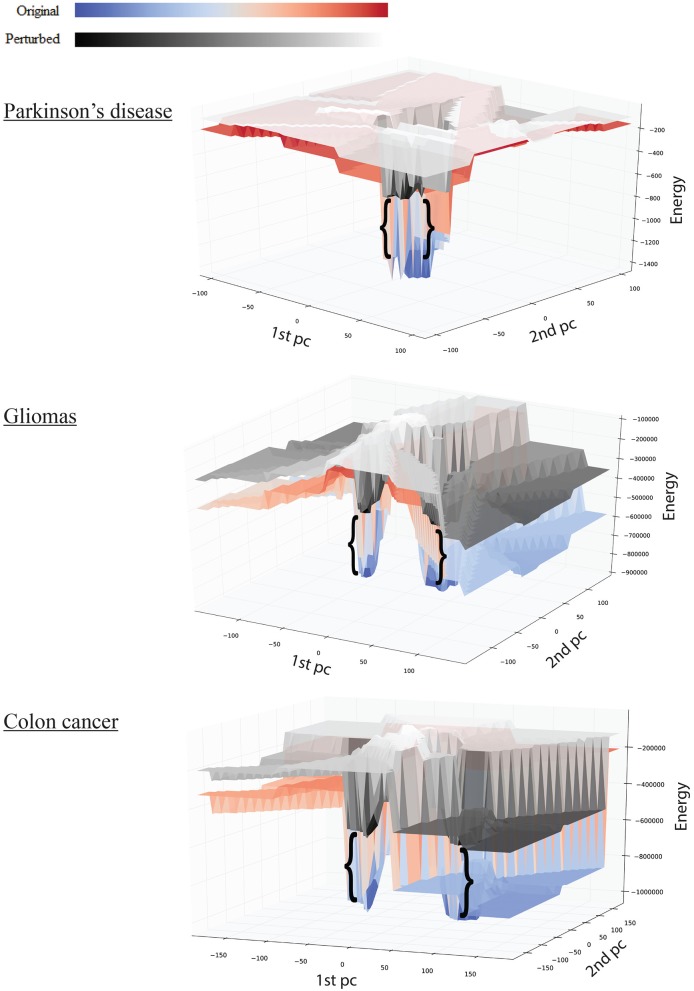
**Effect of perturbations on the shape of the landscape: top, Parkinson's disease; middle, gliomas; bottom panel, colon cancer**. The red-blue surface shows the original network, while the gray-scale surface shows the landscape after perturbation. The brackets show the difference in attractor *E*-value before and after perturbation.

Our proposed model provides a framework to analyse the overall behavior of a GRN during progression from a normal to a disease state. Figure 2 shows a 3D view of the Hopfield energy landscape constructed from each of our three datasets, while Figure 3 shows the effect of perturbations on the shape of these landscapes. For each disease-specific correlation network, and for the respective normal and disease attractor networks, we identify the Gene Ontology (GO) biological process (BP) terms and functions, and the genes uniquely associated with each network (Figure 1). The identities of proteins with unique interactions in each grade-specific network are presented in Table S2.

### Case study 1: Parkinson's disease (PD) progression

The first dataset (GSE62283) consists of 45 samples and 9,480 human protein-probes (Table S1), of which 118 probes remain after feature selection (refer to Datasets in section Materials and Methods). Normal samples exhibit the highest average *E*-value (E_normal_ = −120); the energy values are similar between normal and Parkinson's disease samples (E_EPD_ = −122), but the energy decreases substantially at mid-stage PD (E_*PD*_ = −278; Table 1). The energy difference between normal samples before convergence and the normal attractor (ΔE = 1,329) is greater than that between advanced-stage PD samples and the disease attractor (ΔE = 1,173), i.e., the normal attractor is deeper (Table 1); and the average pairwise intra-group distance is greater for the normal attractor (1.72) than for the one representing PD cases (1.52), i.e., the normal attractor is wider as well.

**Table 1:** **Mean energy of samples in their original state i.e., before convergence**.

**Data**	**Group**	***E***	***E* dis. attract^†^**
Parkinson disease	Normal	−120	1329
	Early PD	−122	1329
	PD	−278	1173
Glioma	Normal	−536,922	332,579
	Grade II	−240,789	628,712
	Grade III	−306,800	562,701
	Grade IV	−398,300	471,201
Colon cancer	Normal	−786,802	258,735
	Non-metastatic	−197,287	848,250
	Metastatic	−160,845	884,692

† *Energy-value distance from attractor*.

**Figure 4:**
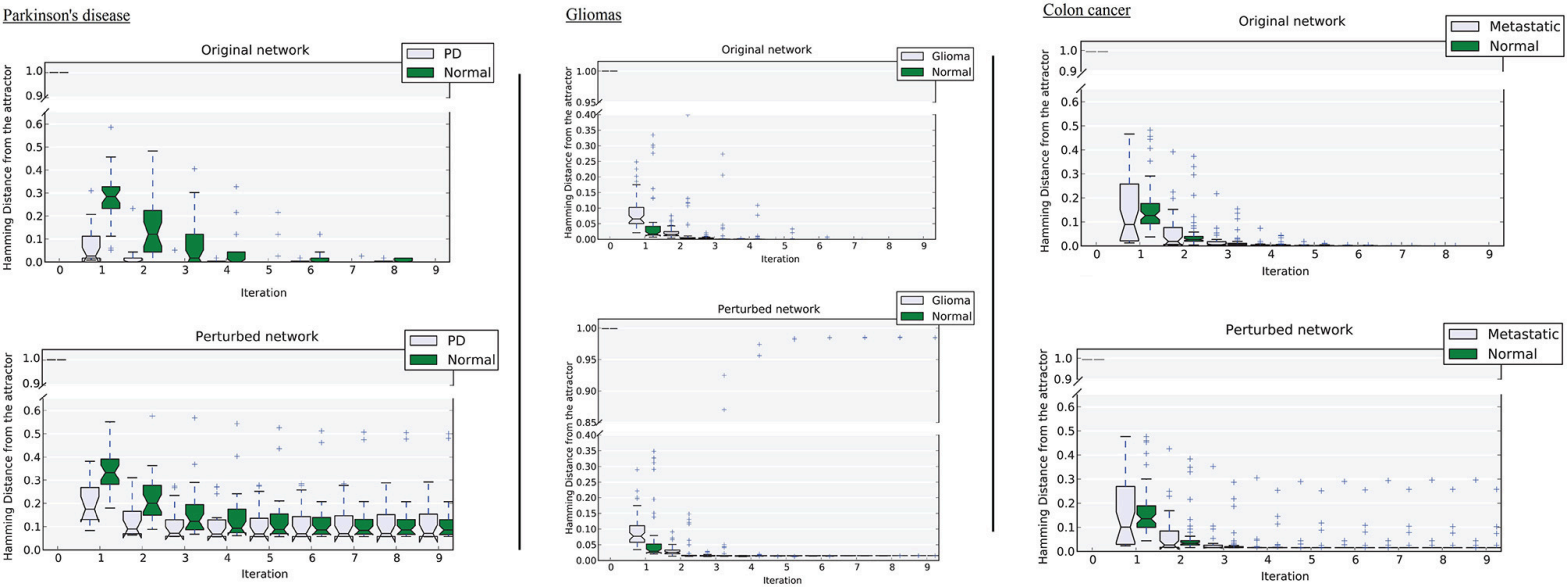
**Distance of samples from their respective attractors at each iteration in the original network, and the perturbed network at 50% perturbation rate**. Left, Parkinson's disease; middle, gliomas; right panel, colon cancer. For each disease-normal pair, comparing the median-line and boxes shows how the attractors differ in size.

We tested the robustness of each attractor by randomly perturbing a large proportion (here 50%) of the edges in *W*, then allowing each sample to relax to its attractor. We then counted the number of samples that did not converge. For the PD data, the normal attractor was more resistant to perturbation: 20% of samples failed to converge, whereas 30% of the corresponding samples did not converge to the disease attractor (Figure 4 and Figure S1).

In this case study, we used all feature-selected protein-probes to construct the stage-specific networks. The correlation network of the PD group had the greatest number of interactions (598 edges), followed by the EPD (186) and normal networks (123). These networks do not have any interactions in common. Node number varied in the unique networks: the PD group had the greatest number of nodes, followed by the normal and EPD groups. Table 2 shows the numbers of positively and negatively co-regulated interactions, and the number of unique interactions needed for the network to progress from one state to the next.

**Table 2:** **Comparison of group-based correlation networks: first 100 feature-selected genes with *p* ≤ 0.0001**.

**Data**	**Group**	**Original network**		**Unique network**	
		**Nodes**	**Edges (±)**	Nodes^*^	**Edges (±)**
Parkinson disease	Normal	118	123 (91/32)	65	123 (91/32)
	Early PD	118	186 (173/13)	61	186 (173/13)
	PD	118	598 (367/231)	96	598 (367/231)
Glioma	Normal	100	1361 (1319/42)	68	1273 (1256/17)
	Grade II	100	1144 (1090/54)	83	1056 (1027/29)
	Grade III	100	101 (75/26)	18	13 (12/1)
	Grade IV	100	988 (906/82)	84	900 (843/57)
Colon cancer	Normal	100	215 (131/84)	47	86 (58/28)
	Non-metastatic	100	200 (113/87)	58	71 (40/31)
	Metastatic	100	139 (81/58)	18	10 (8/2)

* *The genes that remained with no interaction after the filtering process are removed from the network*.

The genes encoding proteins with unique interactions in the normal correlation network were slightly enriched for the GO term *receptor-mediated endocytosis* (*p* = 1.3E-2; Table 3). Among the unique genes associated with this network is nucleoside diphosphate kinase 7 (NME7). The protein encoded by this gene has a major role in synthesis of nucleoside triphosphates other than ATP. Garcia-Esparcia et al. (2015), investigating the effect of NME7 and other genes involved in purine metabolism in PD, interpreted the down-regulation of these genes (mainly expressed in neurons) in the midbrain as a consequence of dopaminergic cell death in PD (Garcia-Esparcia et al., 2015).

**Table 3:** Functional analysis of group-based correlation networks (based on genes with unique interactions in each network)^*^.

**Data**	**Group**	**GO term**	***p*-value**
Parkinson disease	Normal	Receptor-mediated endocytosis	1.3E-2
	Early PD	Positive regulation of calcium-mediated signaling	5.2E-2
	PD	Histone H4-K12 acetylation	2.6E-2
Glioma	Normal	Transmission of nerve impulse	1.0E-5
	Grade II	Gamma-aminobutyric acid signaling	4.9E-5
	Grade III	Neurological system process	1.0E-3
	Grade IV	Synaptic transmission	1.9E-5
Colon cancer	Normal	Ion transport	9.6E-2
	Non-metastatic	Multicellular organismal process	2.7E-2
	Metastatic	Development process	3.2E-2

* *Please refer to Table S4 for the full list of GO terms (p ≤ 0.0001)*.

*Positive regulation of calcium-mediated signaling* (*p* = 5.2E-2) was associated with genes in the EPD correlation network (Table 3). Chemokine C-C motif ligand 17 (CCL17) is one of the unique genes present in this network. Chemokines and their receptors are expressed in cells of the central nervous system and are involved in synaptic transmission. Studies have confirmed their role in neurological diseases such as Alzheimer's and PD, making them potential therapeutic targets (Mines et al., 2007).

The GO term *Histone H4-K12 acetylation* (*p* = 2.6E-2) was associated with genes in advanced PD (Table 3 and Table S4). Among the unique genes present in the network is the FYVE-finger-containing phosphoinositide kinase PIKfyve. The protein encoded by this gene regulates endosome and lysosome function by internalizing and degrading voltage-gated Ca2+ channels (VGCCs). Knockdown of PIKfyve prevents the degradation of VGCCs and thus Ca^2+^ overload and excitotoxicity in neurons, which have been found to play an important role in PD (Tsuruta et al., 2009).

Genes in the normal attractor network were associated with *amino acid metabolism* (*p* = 2.7E-4) and *Nur77 signaling in T lymphocytes* (*p* = 7.1E-5) canonical pathways. The genes in the disease attractor network were enriched for *carbohydrate metabolism* (*p* = 3.1E-3) and associated with the *calcium-induced T lymphocyte apoptosis* pathway (*p* = 1.2E-4; Table S3). A progressive loss of dopaminergic neurons is the main cause of Parkinson's disease; and different types of programmed cell death and signaling pathways and mitochondrial fragmentation have been associated with PD (Venderova and Park, 2012).

### Case study 2: grade-based progression of gliomas

The second dataset (GSE4290) includes 96 samples and 54,613 probes, of which 2,859 remain after feature selection (Table S1). Normal samples in their original state (i.e., before convergence) have the lowest energy value (*E*-value) E_normal_ = −536,922, indicating tighter correlation between the genes in the underlying network. We observe an increase in *E*-value for grades II and III gliomas (E_G2_ = −240,789, E_G3_ = −306,800) and a lower *E*-value for the final stage of the disease E_G4_ = −398,300 (Table 1). The energy difference between samples before and after convergence was greater for the cancer attractor (ΔE = 471,201) than for the normal attractor (ΔE = 332,579), implying that the cancer attractor is deeper than the normal one (Table 1). The average intra-group distance is greater for the cancer (1.79) than for the normal attractor (1.56), suggesting that the former can attract a more-heterogeneous set of samples.

Upon perturbation the cancer attractor is robust, as samples continue to converge even after perturbation of 50% of the edges in the underlying network. This contrasts with the normal attractor, for which the same level of perturbation results in 20% of the samples failing to converge to the corresponding attractor (Figure 4).

Analysis of the underlying correlation networks shows that the normal-stage network has the highest number of significant (*p* ≤ 0.0001) interactions (1,361 edges), followed by the grade II, IV, and III networks. Next, we filtered out common interactions (88 common edges) from the original networks, yielding networks of genes with unique interactions. The normal network had the highest number of unique interactions (1,273 unique edges) followed by grades II, IV, and III (Table 2).

For the normal group, the genes with unique interactions are enriched for GO terms including *transmission of nerve impulse* (*p* = 1.0E-5; Table 3 and Table S4). One of these is neurexin 3 (NRXN3). The protein encoded by NRXN3 functions as a receptor and cell-adhesion molecule in the nervous system. NRXN3 is highly expressed in normal tissues and down-regulated in human gliomas; moreover, Sun et al. (2013) found that Forkhead box Q1 (FoxQ1) promotes glioma proliferation by down-regulating NRXN3, suggesting that it may be a tumor suppressor gene (Sun et al., 2013).

*Gamma-aminobutyric acid (GABA) signaling* (*p* = 4.9E-5) was associated with genes in grade II gliomas (Table 3 and Table S4). GABA is the main inhibitory neurotransmitter in the central nervous system, and has been shown to regulate the growth of many cell types including neuronal and tumor stem cells. GABA response is associated only with low-grade gliomas, suggesting that its absence results in unlimited growth of malignant gliomas (Smits et al., 2012). MET proto-oncogene tyrosine kinase (MET) was among the genes with unique interactions in this network. Following activation by hepatocyte growth factor (HGF) ligand, it triggers cascades of signaling pathways including RAS-ERK and PI3 kinase-AKT. Mutation in MET is associated with tumor growth, angiogenesis and metastasis. Amplification of MET and its ligand HGF has been associated with primary and lower-grade gliomas (Fischer et al., 1997; Beroukhim et al., 2007).

Genes in the grade III correlation network were enriched for *neurological system process* (*p* = 1.0E-3; Table 3 and Table S4). Neurotensin receptor 2 (NTSR2), which encodes a G-protein-coupled receptor, was among the genes with unique interactions in this network. Neurotensin and its receptors including NTSR2 play an important role in oncogenic progression of cancer malignancies. Activated NTSR2 is the key regulatory component that promotes the phosphorylation of extracellular signal-regulated kinase 1/2 (ERK1/2) in glioma cells. ERK1/2 can mediate cell proliferation and apoptosis through overexpression of PDGFRA—a type of receptor tyrosine kinase with ERK-dependant activity (Chen et al., 2014; Ouyang et al., 2015).

*Synaptic transmission* (*p* = 1.9E-5) is one of the GO terms associated with grade IV tumors (Table 3 and Table S4). Among the genes with unique interactions in this group is epidermal growth factor receptor (EGFR), which encodes a member of the protein kinase superfamily. Following receptor activation by binding to a ligand, a series of signaling cascades initiates and drives many cellular responses including cell proliferation and an anti-apoptosis process. EGFR is associated with higher-grade gliomas (Kunkle et al., 2013), and its amplification and activating mutation can be accurate molecular markers in glioma subtyping (Brennan et al., 2009).

We performed similar analyses for the samples that converged to the normal and the cancer attractors. *GABA receptor signaling* (*p* = 2.7E-05) was among the canonical pathways associated with the samples converged to the cancer attractor. *Wnt/catenin signaling*, associated with the normal group (*p* = 1.1E-2), plays an important role in glioma proliferation and tumor progression (Nager et al., 2012; Chen et al., 2013). *Cell death and survival* (*p* = 1.99E-04) was enriched for the cancer attractor, whereas *cellular growth and proliferation* (*p* = 1.36E-4) was among the molecular cellular functions associated with the normal attractor (Table S3).

### Case study 3: colon cancer progression from normal to metastatic state

The third dataset (GSE18105) contains a total of 94 samples and 54,675 probes (Table S1) of which 3,960 probes remained after feature selection. Normal samples at their original state reside at the E_normal_ = −786,802 energy level. Non-metastatic and metastatic samples energy values were E_non−metastatic_ = −197,287 and E_metastatic_ = −160,845, respectively (Table 1). Analysis of the depth of attractors showed that the metastatic attractor is deeper than the normal attractor as the energy difference of samples before and after convergence is higher in the cancer attractor (metastatic vs. normal attractor = 884,692 vs. 258,735; Table 1). Estimates of the size of attractor indicated that the cancer attractor has a greater width, as the average pair-wise Euclidean distance of all samples is larger than the normal attractor (cancer vs. normal = 1.73 vs. 1.69). Perturbation analysis revealed that both attractors are equally robust, as in both cases 10% of the samples do not converge to their respective attractors (Figure 3).

The correlation network analysis showed that the normal network had the highest number of edges (215), followed by the non-metastatic and then metastatic correlation networks with 200 and 139 edges respectively. After removing the common edges, the normal correlation network exhibited 47 nodes and 86 unique edges, followed by the non-metastatic (58 nodes and 71 edges) and the metastatic correlation network (18 nodes and 10 edges) with the fewest genes and unique interactions (Table 2).

The genes with unique interactions in the normal network were enriched for *ion transport* (*p* = 9.6E-2; Table 3 and Table S4). Prostate cancer susceptibility candidate (PRAC) is among the unique genes in this network. It is specifically expressed in the human prostate, rectum and distal colon and has been shown to have a regulatory role in the nucleus (Liu et al., 2001).

The non-metastatic network was enriched for *multicellular organismal process* (*p* = 2.7E-2; Table 3 and Table S4) including the calcium activated chloride channel A1 (CLCA1). It is expressed mainly in the colon, intestine, and appendix. CLCA1 plays a role in tumor suppression and has been shown to have a down-regulated expression in colorectal cancer (Yang et al., 2013).

The genes in the metastatic network were associated with *developmental process* (*p* = 3.2E-2; Table 3 and Table S4). Among the unique genes in this group is IGF2BP3, a member of mRNA protein binding family and an oncofetal protein that regulates expression of genes involved in tumor cell proliferation, chemo-resistance and metastasis. *In vitro* studies have revealed that IGF2BP3 up-regulated expression enhances tumor growth, drug-resistance and metastasis in various human cancers (Lederer et al., 2014).

The genes with unique interactions in the normal attractor correlation network were enriched for *B cell development* pathways (*p* = 3.5E-3) and *cell morphology* (*p* = 1.06E-5) for molecular and cellular function. In the case of the cancer attractor correlation network, the genes were enriched for *cellular movement* (*p* = 2.0E-4) for cellular function and associated with the *autoimmune thyroid disease signaling* pathway (*p* = 3.6E-3; Table S3). Thyroid hormone signaling has been shown to be a major factor in digestive system growth, and homeostasis and the expression of thyroid-hormone receptors has been associated with colon cancer progression. Other studies have suggested that thyroid-hormone signaling may suppress colon cancer invasiveness (Brown et al., 2013).

## Discussion

Large-scale multi-omics studies have been carried out to investigate the molecular dynamics underlie complex disease progression. For instance, in a recent study Cho et al. (2016) used Boolean networks to construct the attractor landscape of colorectal tumorigenesis from previously published canonical signaling pathways. Combined with already known mutation data and prior knowledge of signaling networks in cancer, their model provides a new approach for discovering novel therapeutic targets for cancer patients (Cho et al., 2016). Parsons et al. (2008) provided a genetic landscape of glioblastomas by integrating mutations and copy number alternations by sequencing 20,661 protein-coding genes, and performed gene expression analysis in 22 human tumor samples. They inferred key genes associated with glioblastoma including IDH1, and showcased the potential of genome-wide genetic studies in opening novel avenues in brain cancer research (Parsons et al., 2008).

Attractor landscape models provide a quantitative approach to understand the dynamics of GRNs during development and disease formation. Here we construct disease-progression landscape models using the mathematical framework of Hopfield networks. We used three datasets to capture different aspects of this process: the advancement of Parkinson's disease from normal to an advanced state, the progression of glioma from normal to grade IV, and the progression from normal colon to metastatic colon cancer. For each, we mapped these different stages of disease progression to HN energy profiles. Attractors in these landscapes correspond to the initial (normal) state of the cell, and the (potentially) final stage of the disease. Each landscape is constructed using the correlation network among all feature-selected protein- or gene-pairs in the microarray data, reflecting the biological activity and changes in the GRN during disease progression. We take a network approach to identify unique molecular interactions at each disease stage, and examine their biological features.

Complex disease can often be attributed to inappropriate regulatory signals, together with accumulation of genetic mutations. It is important to identify specific genes whose mutation results in moving cells from normal to a disease state, and eventually to disease progression, characterized in cancer by acquisition of metastatic potential. With a view of disease as a pre-existing configuration of the GRN that has not been accessed by a normal cell, this model provides a systems view on GRN rewiring during disease formation and progression.

Unlike other computational models of attractor landscapes, our model does not require prior knowledge of the system. Because the Hopfield energy values are computed via a Lyapunov function, they are guaranteed to converge to a local energy minimum that represents a stable state of the network or attractor. In this formalism, the energy of samples at their original state (i.e., before convergence) is inversely proportional to the tightness of the correlations in the network: the more-correlated the expression of feature-selected genes, the lower the energy (Taherian Fard et al., 2016). We emphasize that we do not provide labels for samples (non-parametric learning), and the learning algorithm constructs attractors solely on the similarities among the patterns in the gene-expression data. Samples with similar activity patterns typically converge to the same attractor, but are occasionally misclassified (i.e., converge to a different attractor). The difference between energy values before and after convergence provides an estimate of the depth of the attractor.

As discussed above, computational models of GRNs have their own definition of attractors and trajectories. Here we capture trajectories of disease progression through the energy values of samples before convergence (color-coded in the 3D landscape, Figure 2). Attractors are the result of an artificial updating of the network (the iterative HN process) and do not correspond to actual cell types, but the computed energy of samples at their attractor state provides a quantitative measure of the extent of gene-expression similarity among the samples at the corresponding stage of disease progression. Thus, in the PD case study, we observe that normal and EPD samples converge to the normal attractor, whereas PD samples converge to the disease attractor. By contrast, in both cancer case studies we observed high intra-group heterogeneity within the mid-stage subgroups: a subset of grade II gliomas converge to the normal attractor while the rest converge to the cancer attractor, while a subset of non-metastatic CRC tumors converges to the normal attractor while the rest converge to the disease attractor.

Our results indicate that in the case of PD, the normal attractor has a slightly broader basin of attraction than does the disease attractor, whereas the opposite is true for each of the cancer case studies. Thus, samples extracted from tumors are more heterogeneous than the corresponding normal tissues, whereas there is a relatively narrow basin of attraction in PD. It remains to be seen whether this result will prove general.

A stable attractor is robust to perturbation, such that introducing noise into the network does not affect the fraction of samples converging to that attractor. By contrast, a less-stable attractor does not bear the insult. The biological interpretation is that if the attractor is robust, more molecular changes to the network are required to move cells out of the phenotypic state. The robustness of attractors proved to be specific to each case study, and was influenced by factors including the presence of nodes strongly correlated with many other nodes.

For the HN framework to have utility, the input data must meet certain requirements: at least three time-points must be available, with at least three samples per time-point, and samples must be sufficiently homogeneous. As we use a correlation measure to constitute the edges, a larger sample size provides a stronger signal for the gene-activity pattern. To capture the dynamics of GRN at well-defined stages of disease progression, we chose publicly available datasets with different characteristics. Thus, in the first case study we focused on severity of disease by using human protein microarray data from patients with early- and advanced-stage PD. In the second case study, we used gene-expression profiles of patients with different cancer grades. We utilized the graded glioma samples, assuming that normal and lower-grade gliomas are precursors of higher-grade gliomas. Similarly in the third case study, we targeted the metastatic process in colon cancer, assuming a progression from normal to non-metastatic disease to metastasis.

Our HN landscape model, with a simple computational workflow and requiring minimum prior knowledge of the underlying network, provides a framework to study the process of disease formation and progression, and identifies genes that are potential key drivers of this process. The feature-selected gene sets are informative on the disease states *per se*, while still supported by the evidence we present in this paper that the genes with unique interactions at each stage-specific network of the disease are the potential key drivers of disease progression. We show that by applying this framework to appropriate disease stage-specific protein or gene microarray datasets, biologically useful insights can be revealed from the dynamics of the GRN, including the identification of genes and their stage-specific interactions that are involved in disease progression. In constructing GRN models, it could be desirable to include data on other molecular processes that are also involved in driving the cell toward a specific fate, e.g., histone modifications, copy-number variation, and time-course proteomics data; or in the context of disease, specific mutations and their downstream regulatory consequences. The Hopfield model framework is sufficiently flexible to utilize and integrate a variety of molecular data, so long as at least two states or conditions are present; input data need not be quantitative, e.g., could represent the presence or absence of a histone mark. Combining all layers of information will allow more-accurate and detailed modeling of these dynamic systems of development or disease.

## Author contributions

AT and MR conceptualized the research; AT designed and performed the analysis; AT and MR wrote the manuscript. Both authors approve its submission for publication.

## Funding

Funding was provided by Australian Research Council award DP110103384 to MR. This project was supported in part by strategic funds from The University of Queensland.

### Conflict of interest statement

The authors declare that the research was conducted in the absence of any commercial or financial relationships that could be construed as a potential conflict of interest.
