# Supplementary material for: Modeling the Attractor Landscape of Disease Progression: a Network-Based Approach
Source: Front Genet. 2017 Apr 18;8:48. doi: 10.3389/fgene.2017.00048 (PMC5394169; doi:10.3389/fgene.2017.00048)
Supplement: Supplementary file 1 [file Table1.DOCX]

**Supplementary Table 1**

| Dataset | Microarray platform | # of samples | Groups | # of probes | # feature selected probes |
| --- | --- | --- | --- | --- | --- |
| Glioma  (GSE4290) | Affymetrix Human Genome U133 Plus 2.0 Array | 96 | Normal X 23 | 54,613 | 2,859 |
|  |  |  | Grade II X 38 |  |  |
|  |  |  | Grade III X 12 |  |  |
|  |  |  | Grade IV X 23 |  |  |
| Colon cancer  (GSE18105) | Affymetrix Human Genome U133 Plus 2.0 Array | 94 | Normal X17 | 54,675 | 3,960 |
|  |  |  | Non metastatic X 47 |  |  |
|  |  |  | Metastatic X 30 |  |  |
| Parkinson disease  (GSE62283) | Invitrogen ProtoArray v5.0 | 45 | Normal X 15 | 9,480 | 119 |
|  |  |  | Early PD X 15 |  |  |
|  |  |  | PD X 15 |  |  |

Table S1: Datasets.
