# Supplementary material for: Modeling the Attractor Landscape of Disease Progression: a Network-Based Approach
Source: Front Genet. 2017 Apr 18;8:48. doi: 10.3389/fgene.2017.00048 (PMC5394169; doi:10.3389/fgene.2017.00048)
Supplement: Supplementary file 3 [file Table3.DOCX]

**Supplementary Table S3**

Table S3: Biological properties of attractors: based on genes with unique interactions extracted from the correlation network with *p-value* $\leq$ 0.0001.

| Dataset | Attractor | Canonical pathways | *p-value* | Molecular and cellular functions | *p-value* |
| --- | --- | --- | --- | --- | --- |
| Parkinson disease | Normal | Nur77 signalling in T lymphocytes | 7.1E-5 | Amino acid metabolism | 2.7E-4 |
|  | Disease | Calcium-induced T lymphocyte apoptosis | 1.2E-4 | Carbohydrate metabolism | 3.1E-3 |
| Glioma | Normal | Wnt/-catenin signalling | 1.1E-2 | Cellular growth and proliferation | 1.3E-4 |
|  | Cancer | GABA receptor signalling | 2.7E-5 | Cell death and survival | 1.9E-7 |
| Colon cancer | Normal | B cell development | 3.5E-3 | Cell morphology | 1.0E-5 |
|  | Cancer | Autoimmune thyroid disease signalling | 3.6E-3 | Cellular movement | 2.0E-4 |
