# Supplementary material for: Modeling the Attractor Landscape of Disease Progression: a Network-Based Approach
Source: Front Genet. 2017 Apr 18;8:48. doi: 10.3389/fgene.2017.00048 (PMC5394169; doi:10.3389/fgene.2017.00048)
Supplement: Supplementary file 4 [file Table4.DOCX]

**Supplementary Table S4**

Table S4: Functional analysis of group-based correlation networks (based on genes with unique interactions in each network) with *p-value* $\leq$ 0.0001.

| Data | Group | GO term | p-value |
| --- | --- | --- | --- |
| Parkinson disease | Normal | Receptor-mediated endocytosis  4-aminobutyrate Degradation I  Glutamate Receptor Signaling  Nur77 Signaling in T Lymphocytes  Glutamate Degradation III (via 4-aminobutyrate)  Calcium-induced T Lymphocyte Apoptosis | 1.3E-2  8.36E-3  1.11E-2  1.19E-2  1.39E-2  1.47E-2 |
|  | Early PD | Positive regulation of calcium-mediated signalling  iCOS-iCOSL Signaling in T Helper Cells  CD28 Signaling in T Helper Cells  Calcium-induced T Lymphocyte Apoptosis  CTLA4 Signaling in Cytotoxic T Lymphocytes  PKC Signaling in T Lymphocytes | 5.2E-2  3.31E-4  4.33E-4  7.57E-4  2.44E-3  5.46E-3 |
|  | PD | Histone H4-K12 acetylation  Nur77 Signaling in T Lymphocytes  Calcium-induced T Lymphocyte Apoptosis  Tyrosine Biosynthesis IV  4-aminobutyrate Degradation I  iCOS-iCOSL Signaling in T Helper Cells | 2.6E-2  1.80E-3  2.48E-3  1.22E-2  1.22E-2  1.36E-2 |
| Glioma | Normal | Transmission of nerve impulse  GABA Receptor Signaling  TCA Cycle II (Eukaryotic)  Regulation of eIF4 and p70S6K Signaling  Neuroprotective Role of THOP1 in Alzheimer's Disease  cAMP-mediated signaling | 1.0E-5  6.40E-7  5.52E-2  5.72E-2  9.40E-2  1.03E-1 |
|  | Grade II | Gamma-aminobutyric acid signalling  GABA Receptor Signaling  Wnt/-catenin Signaling  Cholecystokinin/Gastrin-mediated Signaling  Glioma Signaling  Pyrimidine Deoxyribonucleotides De Novo Biosynthesis I | 4.9E-5  1.21E-6  1.18E-2  3.25E-2  3.80E-2  6.24E-2 |
|  | Grade III | Neurological system process  GABA Receptor Signaling  Hepatic Fibrosis / Hepatic Stellate Cell Activation  Cholecystokinin/Gastrin-mediated Signaling  Glioma Signaling  Natural Killer Cell Signaling | 1.0E-3  1.21E-6  1.46E-2  3.25E-2  3.80E-2  4.58E-2 |
|  | Grade IV | Synaptic transmission  GABA Receptor Signaling  Hepatic Fibrosis / Hepatic Stellate Cell Activation  Cholecystokinin/Gastrin-mediated Signaling  Glioma Signaling  Natural Killer Cell Signaling | 1.9E-5  1.82E-6  1.81E-2  3.77E-2  4.40E-2  5.29E-2 |
| Colon cancer | Normal | Ion transport  Nicotine Degradation III  Melatonin Degradation I  Nicotine Degradation II  Ephrin B Signaling | 9.6E-2  4.48E-3  5.86E-3  6.05E-3  8.04E-3 |
|  | Non metastatic | Multicellular organismal process  Atherosclerosis Signaling  Tyrosine Biosynthesis IV  Phenylalanine Degradation I (Aerobic)  Sucrose Degradation V (Mammalian) | 2.7E-2  1.82E-4  6.66E-3  8.87E-3  1.99E-2 |
|  | Metastatic | Development process  Atherosclerosis Signaling  Fatty Acid Activation  Mitochondrial L-carnitine Shuttle Pathway  Intrinsic Prothrombin Activation Pathway | 3.2E-2  3.12E-03  8.59E-03  1.12E-02  1.91E-02 |
