# Supplementary figures and images for: Modeling the Attractor Landscape of Disease Progression: a Network-Based Approach
Source: Front Genet. 2017 Apr 18;8:48. doi: 10.3389/fgene.2017.00048 (PMC5394169; doi:10.3389/fgene.2017.00048)

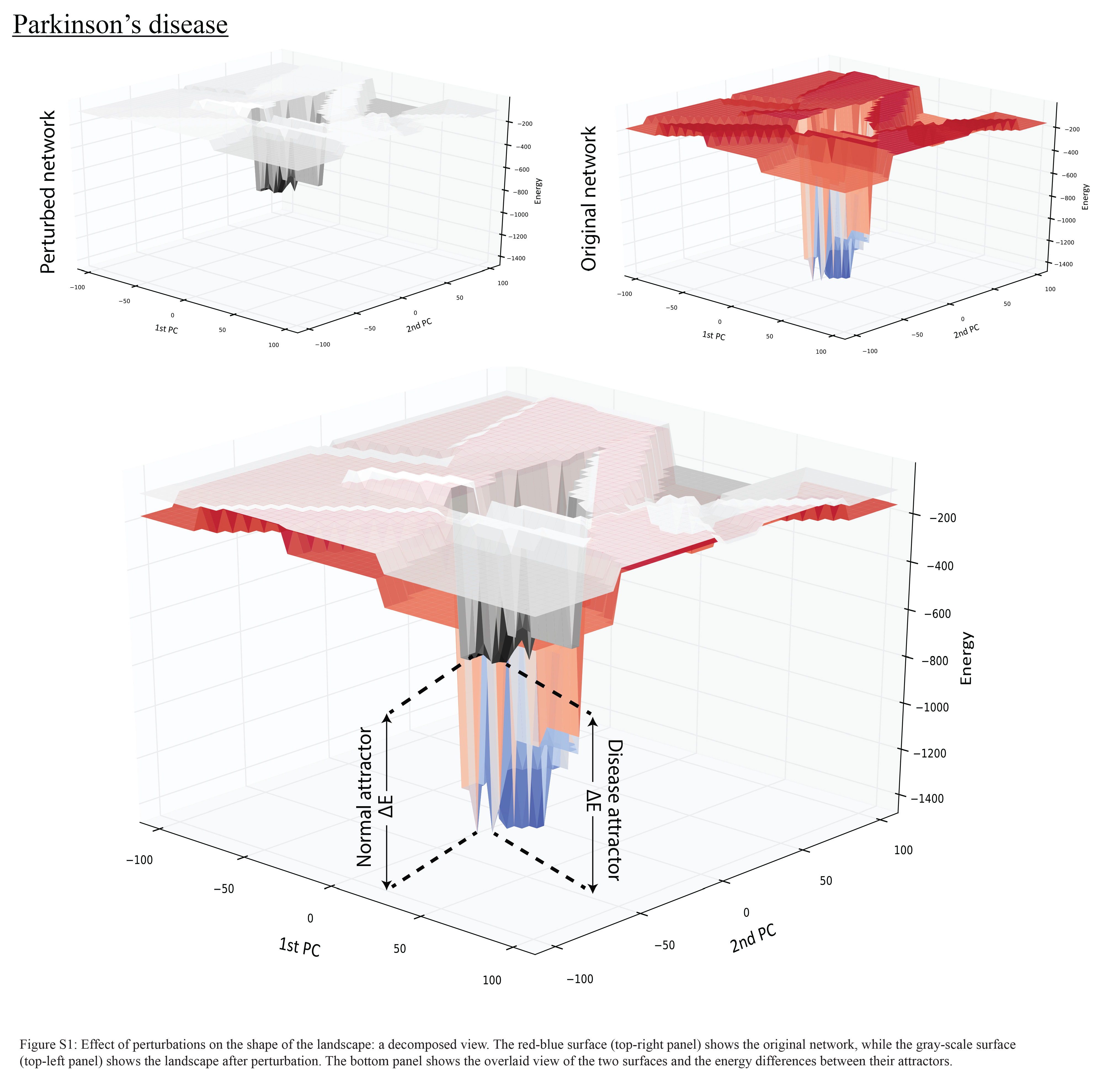

Supplement: Supplementary file 5 [file Image1.jpg]
